# Orthopaedic Wire Debridement: A Novel Surgical Technique for Hypergranulated Burn Wounds

**DOI:** 10.1007/s00268-023-07147-6

**Published:** 2023-09-14

**Authors:** Nathan Carl English, Gary Dos Passos

**Affiliations:** 1 Department of General Surgery, Groote Schuur Hospital, University of Cape Town, Cape Town, South Africa; 2 Division of Plastic, Reconstructive and Maxillo-Facial Surgery, Groote Schuur Hospital, University of Cape Town, Cape Town, South Africa; 3 Burn Unit, Red Cross War Memorial Children’s Hospital, Cape Town, South Africa

## Abstract

The burn wound healing process can be protracted due to various factors, including burn depth, infection and hypergranulation. Hypergranulation impedes epithelialisation macroscopically by preventing cellular migration across the wound bed and microscopically through cell-to-cell signal interferences. Debridement, which is the act of removing necrotic tissue, hypergranulation, slough and foreign debris from the wound in order to expose the underlying viable bed, can be achieved using various techniques. This aids with wound bed preparation to facilitate and expedite healing. In this article, we present a novel surgical debridement technique using a malleable orthopaedic cerclage wire for the management of a hypergranulated burn wound.

**Supplementary Information:**

The online version contains supplementary material available at 10.1007/s00268-023-07147-6.

## Introduction

**Fig. 1:**
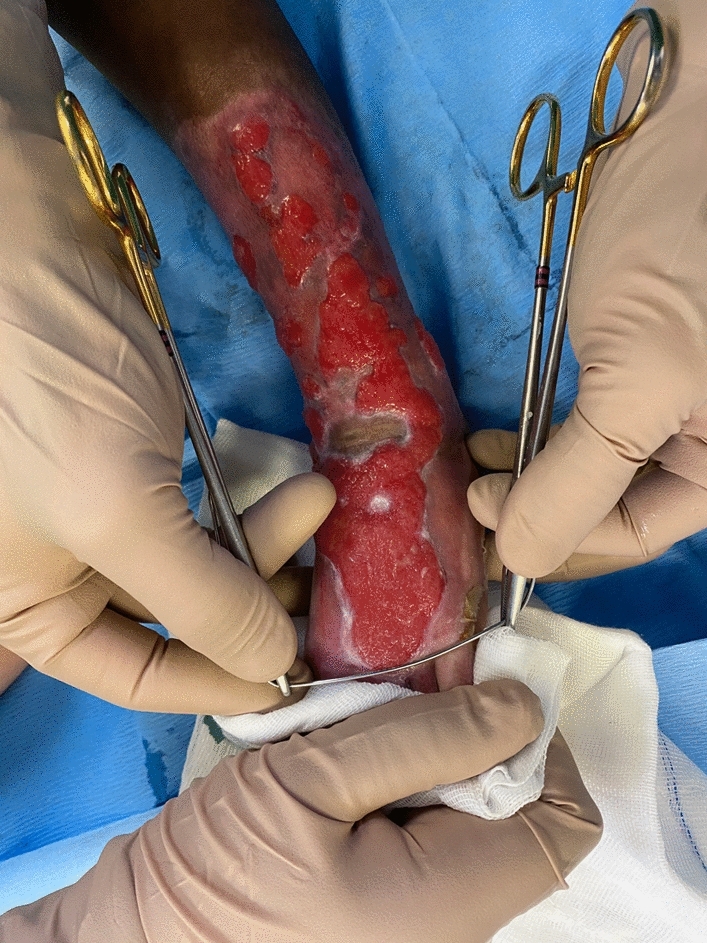
Hypergranulated islands amongst surrounding healed skin

It is commonplace that most superficial to deep partial thickness burns will heal within 3–4 weeks by secondary intention, facilitated by the application of anti-bacterial dressings [1]. However, there are instances where unhealed, hypergranulated islands co-exist amongst the surrounding healed skin, requiring debridement and grafting, as it has been noted to increase healing time and contribute to contractures [2] (Fig. 1).

## The hypergranulated burn wound

The mass of capillaries, inflammatory cells, fibroblasts and extra-cellular matrix formed at the site of injury, collectively known as granulation tissue, ensues during the proliferative (reparative) phase of tissue healing [3]. The objective of its formation is to re-establish the structural integrity at the site of injury and provide a latticework along which epithelialisation can occur. When this process is overwhelmed, the wound is filled with an excess of granulation tissue beyond the amount required to fill the defect, resulting in an area of hypergranulation. Clinically, it is recognised as a pink-reddish friable mass, with a shiny surface, extruding above the level of the surrounding healed skin. Whilst the aetiology of hypergranulation has not been comprehensively described, predisposing factors include excessive wound exudate, prolonged foreign body irritation and external friction [2], as well as protracted stimulation of fibroplasia and angiogenesis. The mechanism by which it impedes epithelialisation is multi-modal. Macroscopically, it acts as a mechanical barrier preventing keratinocyte migration across the wound bed, whilst surface biofilm, made up of microscopic colonies of bacteria dispersed within an extra-cellular polymeric substance (EPS) [4], drives a low-grade inflammatory process which alters signalling pathways between keratinocytes and various growth factors, thereby inhibiting wound closure.

## The orthopaedic wire debridement technique

Due to the paucity of randomised controlled trials comparing surgical and non-surgical modalities, the treatment of hypergranulation tissue is a contentious topic in the literature, particularly in the context of burn injuries [2]. Variable success rates have been reported using a number of topical agents including hypertonic saline, silver nitrate, and steroids, whilst sharp surgical excision and ablative laser therapy have also been described. The orthopaedic wire debridement technique is classified as a surgical modality using a malleable 1.25-mm cerclage wire grasped between two ratcheted instruments, such as needle holders. The inherent mechanical properties of the wire contribute to the effectiveness and efficiency of the technique, with a multitude of surgical benefits.

**Fig. 2:**
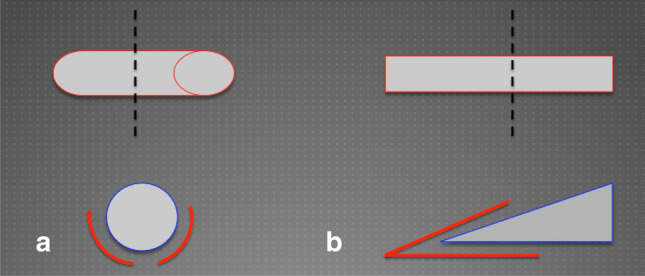
**a** Cross section of orthopaedic cerclage wire cutting edge. **b** Cross section of planar blade cutting edge (e.g. Humby knife blade)

Firstly, in contrast to other sharp, planar instruments, the round body of the wire allows for effective debridement of the friable granulation tissue, whilst being relatively atraumatic to the surrounding healthy skin (Fig. 2).

**Fig. 3:**
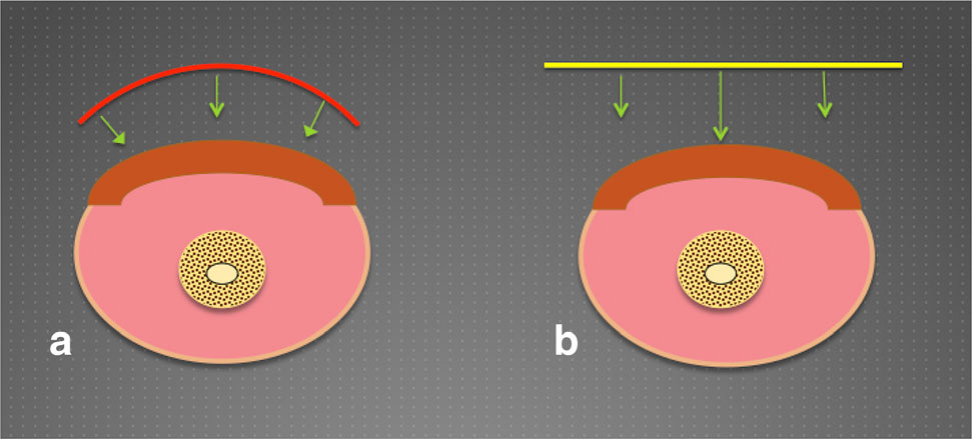
**a** Schematic representation of cerclage wire manipulated to replicate the contour of the body surface, thereby enabling equal distribution of force across the wound bed. **b** Schematic representation of unequal pressure distribution exerted by planar instruments resulting in overzealous debridement in the centre of the wound

Secondly, the malleability of the wire allows for the manipulation of its curvature in order to replicate the contour of the body-surface affected by the hypergranulation, allowing for even distribution of pressure across the wound bed (Fig. 3).

Thirdly, by adjusting the distance between the surgeon's thumbs, the width of the debriding edge can be tailored to the wound. These attributes allow the operator to customise the instrument according to the wound configuration of individual patients, thereby enabling single-pass debridement and effective wound bed preparation for grafting both flat and curved body surfaces.

### Operative steps

**Fig. 4:**
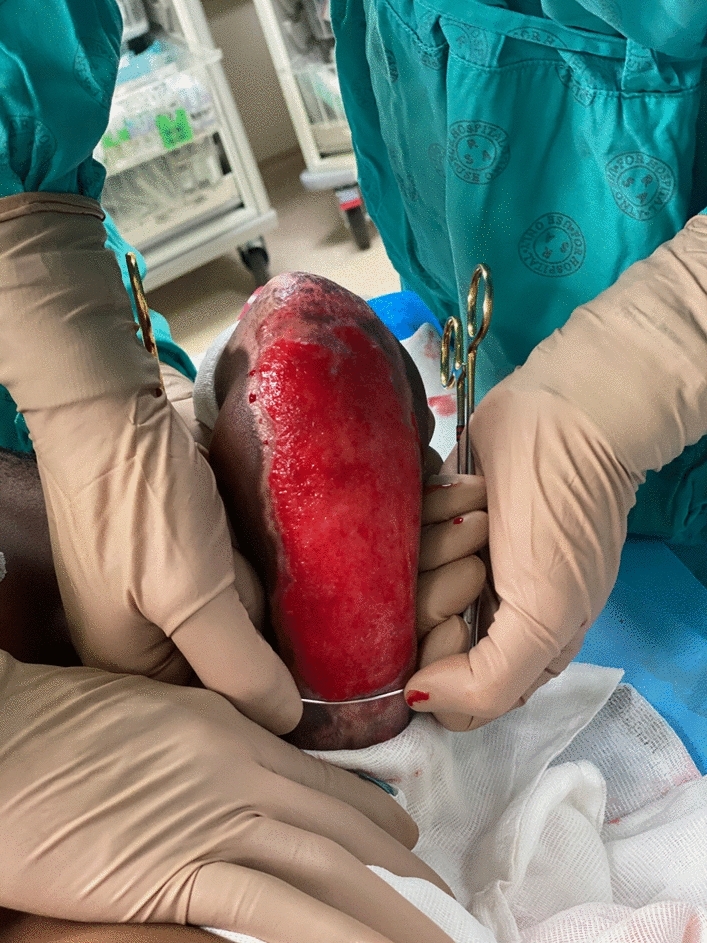
Instrument held at distal wound margin, parallel to the body surface

The first step is to assess the wound configuration. The size, shape and contour are used to set the debriding-edge width (inter-grasper distance) and the curvature of the wire. Once the assembly is complete, the instrument is held at the interface between the distal wound margin and the surrounding healed skin, with the curvature of the wire parallel to the body-surface (Fig. 4).

**Fig. 5:**
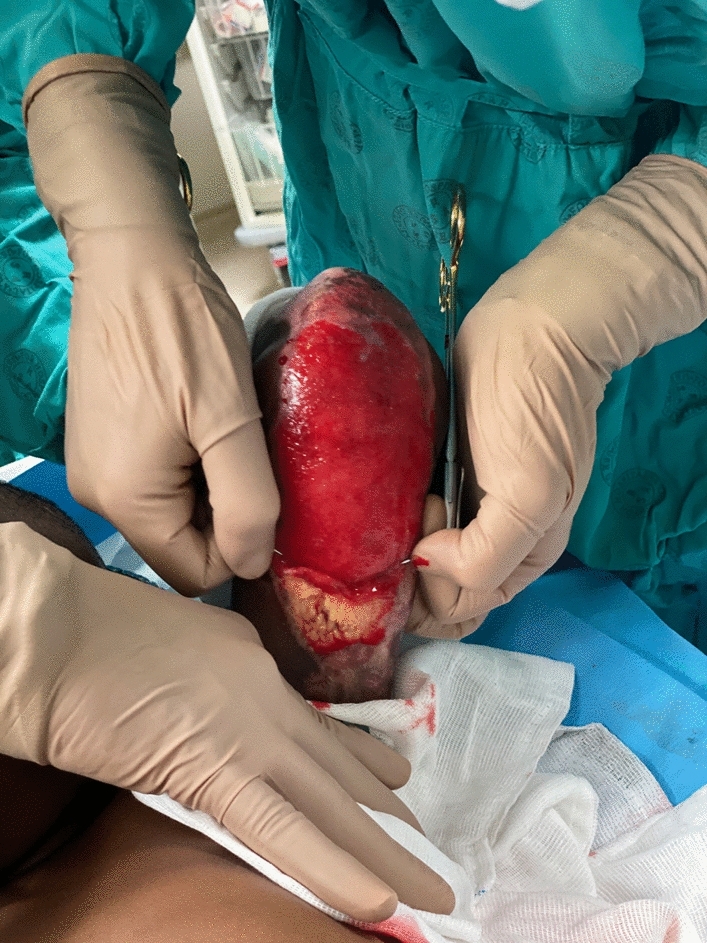
Counter traction applied by assistant, whilst downward pressure is maintained and the instrument being drawn towards the surgeon

With the graspers firmly held, gentle downward pressure is applied by each thumb at the wound edge. This establishes a plane between the hypergranulation tissue and the underlying healthy wound bed. The wire is then gradually drawn towards the surgeon, while the assistant applies countertraction. This results in a single-pass, tangential-type excision (Fig. 5).

**Fig. 6:**
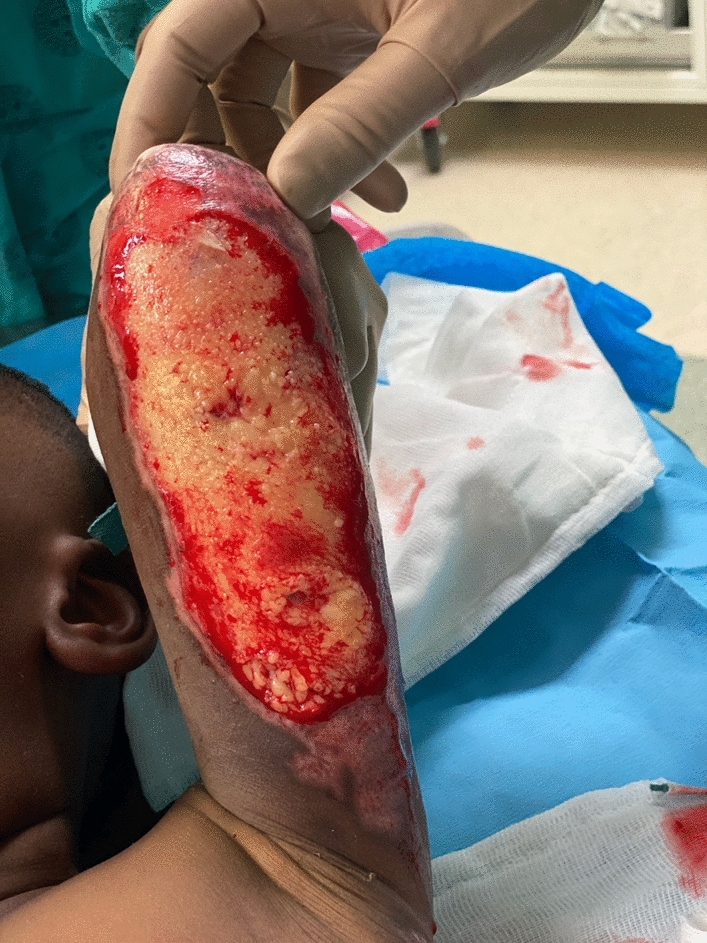
Hypergranulation tissue debrided *en bloc,* with preservation of surrounding healed skin at wound margin circumferentially

When approaching the proximal wound edge, the instrument can be removed from underneath the overlying hypergranulation mass, and repositioned at the interface between the proximal wound margin and surrounding healed skin. The manoeuvre is repeated; however, the instrument is now advanced away from the surgeon along the plane previously established, with an aim to remove the mass of hypergranulation *en bloc*. This prevents trauma to the freshly healed and fragile proximal wound edge. In irregularly shaped wounds, the distance between the surgeon's thumbs can be continually adjusted to accurately debride the hypergranulation tissue, without traumatising healthy healed tissue at the wound margin (Fig. 6).

This technique allows for the removal of large volumes of hypergranulation in a single pass, and we prefer to infiltrate the wound bed with clysis [5] prior to debridement to help control bleeding (Supplementary material).

## Conclusion

Hypergranulation tissue is a deleterious factor resulting in protracted wound healing, thus requiring debridement. The orthopaedic wire technique is beneficial for multiple reasons. It is simple, cost-effective—approximating 1.00 ZAR (0.054 USD) per centimeter of wire, and efficient. It does not require special equipment and is easily reproducible. These attributes make the technique applicable in a busy burns unit, with resource constraints.

### Supplementary Information

Below is the link to the electronic supplementary material.

Supplementary file1 (MP4 21594 KB)
